# Cystic intracranial solitary fibrous tumor: a case report

**DOI:** 10.3389/fonc.2024.1422779

**Published:** 2024-07-02

**Authors:** Yongzhe Li, Dongxue Li, Li Yang, Jiaren Zhang, Xiaoyu Gu, Linfeng Song, Binlin Tian, Tingchao Li, Lin Jiang

**Affiliations:** ^1^ Department of Radiology, The Third Affiliated Hospital of Zunyi Medical University (The First People’s Hospital of Zunyi), Zunyi, China; ^2^ Department of Pathology, The Third Affiliated Hospital of Zunyi Medical University (The First People’s Hospital of Zunyi), Zunyi, China

**Keywords:** tumor, intracranial tumor, solitary fibrous tumor, cystic, magnetic resonance imaging, case report

## Abstract

Solitary fibrous tumor (SFT) is a rare spindle cell tumor originating from mesenchymal tissue, and even rarer when it occurs intracranially. This case report described a 42-year-old man who presented with headache and limb weakness for more than 10 days. Magnetic resonance imaging (MRI) showed a well-defined multicompartmental cystic space-occupying lesion in the left occipital region, with surrounding edema and a compressed left lateral ventricle, the mass growing across the cerebellar vermis, which was initially diagnosed as hemangioblastoma. Neurosurgery was utilized to successfully remove the mass, and intracranial solitary fibrous tumor (ISFT) was identified by postoperative pathological analysis. Here, this article describes the imaging manifestations and pathologic features of a case of cystic intracranial solitary fibrous tumor, aiming to improve the understanding and diagnosis of this disease in order to provide an accurate therapy plan.

## Introduction

Intracranial solitary fibrous tumor is a rare intracranial tumor that was first observed by Carneiro et al, who named it in 1996 (1). Accounting for only 0.09% of intracranial meningeal-related tumors (2). The solitary fibrous tumor that appears cystic on imaging is even rarer. ISFT typically develops in middle-aged and older individuals, and has a slight male predominance (3). The clinical presentation of this tumor is different depending on the duration of the disease and the location of the tumor, and due to its rarity, it is not well known to both physicians and radiologists, resulting in a high rate of misdiagnosis in the preoperative period. A puncture biopsy remains the gold standard for diagnosis. Resection followed by radiotherapy or chemotherapy is the main therapy of choice for ISFT (4, 5). The aim of this article is to use the description of imaging performance to further increase the understanding of cystic intracranial solitary fibrous tumor.

## Case presentation

The patient, a 42-year-old man, was admitted to our hospital with headache, and weakness of both legs for ten days. The patient had a history of a fall 10 years earlier, which was diagnosed at that time as a contusion in the left cerebellar hemisphere and occipital lobe, with softening foci formation. The patient was seen in the outpatient clinic three years ago for paroxysmal dizziness and insomnia, and the cranial CT was suggested that the left cerebellar hemisphere and the left occipital lobe had foci of cerebral softening. The patient was readmitted to the hospital for aggravation of headache, his vital signs were standard, and neurological examination revealed a positive right finger-nose test.

**Figure 1:**
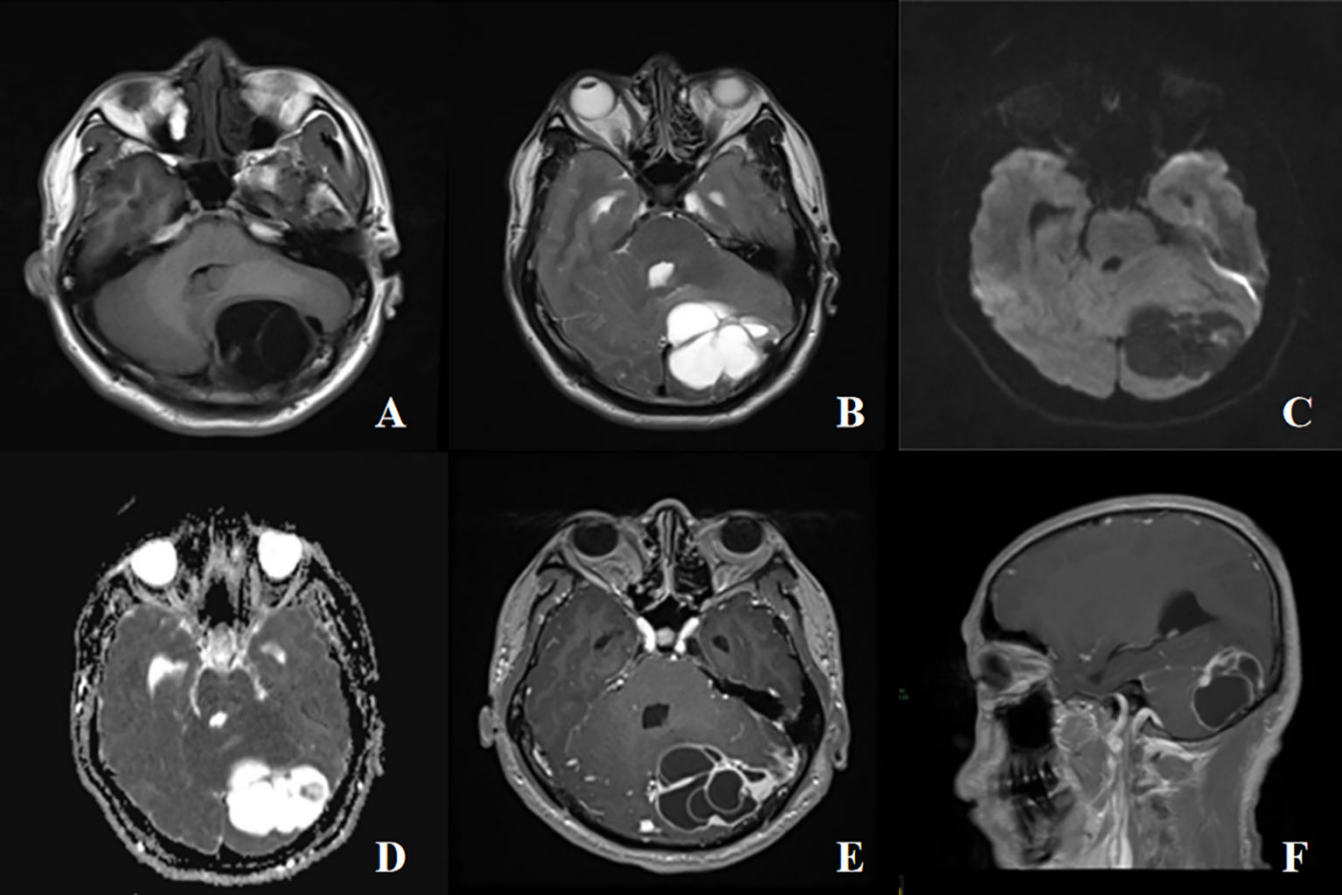
A 42-years-old male with cystic intracranial solitary fibrous tumor. **(A, B)** The mass was multicompartmental cystic with hypointensity T_1_WI and hyperintensity T_2_WI in the cystic component and equal/slightly hyperintensity T_1_WI and mixed signal T_2_WI in the solid component, with surrounding edema, mass effect and compression of the ventriculus quartus cerebri, no obvious destruction of adjacent bone. **(C, D)** A high signal on diffusion-weighted imaging (DWI), and a low signal on apparent diffusion coefficient (ADC). **(E)** The enhanced scan showed that the solid component of the lesion and the cystic wall were significantly enhanced. **(F)** Sagittal view shows the mass growing across the vermis of the cerebellum.

Magnetic Resonance Imaging (MRI) Scanning and Enhancement: there was a mass of predominantly cystic composition in the right occipital region, measuring approximately 5.1×5.3×6.0cm, which crossed the cerebellar peduncle, compressed the adjacent brain parenchyma, and the fourth ventricle was narrowed by the compression (**Figure 1F**). The mass was multicompartmental cystic with hypointensity T_1_WI and hyperintensity T_2_WI in the cystic component and equal/slightly hyperintensity T_1_WI and mixed signal T_2_WI in the solid component (**Figures 1A, B**). Diffusion-weighted imaging (DWI) showed heterogeneous hyperintense with decreased apparent diffusion coefficient (ADC) values (**Figures 1C, D**). The enhanced scan showed that the solid component of the lesion and the cystic wall were significantly enhanced (**Figure 1E**). Magnetic resonance venography (MRV) showed the left transverse sinus to be tiny developed and partially invaded with interrupted continuity. Based on the results of the preoperative examination, hemangioblastoma was suspected and surgical resection with craniotomy was performed. Surgery was conducted in the left lateral position using the left occipital transtentorial approach. The tumor was characterized by a red color, the cystic fluid was a faint yellow color, the tumor had clear boundaries, and the surrounding blood vessels were small and numerous. During the operation, the tumor cavity ruptured and bled for several times, and the mass was completely removed under the microscope and sent to pathology.

**Figure 2:**
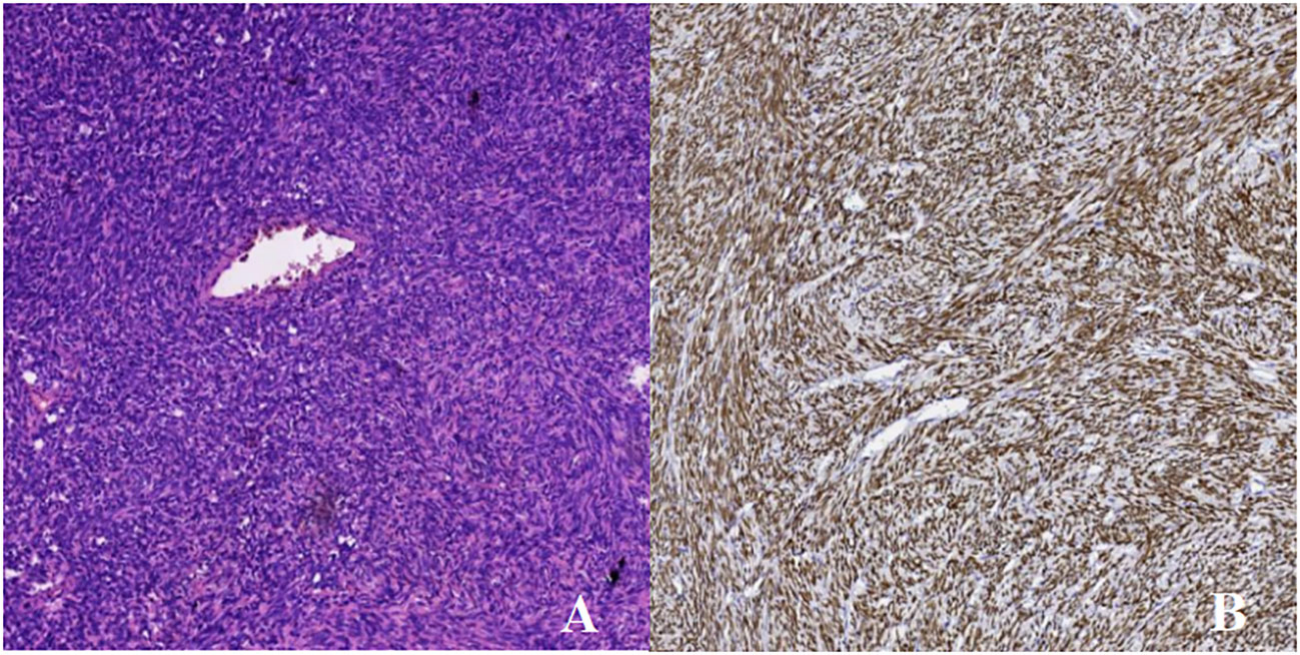
HE staining×100 and immunohistochemistry. **(A)** H&E staining revealed the tumor cells are spindle-shaped, diffusely distributed, the density of tumor cells is high, the pathological mitotic image is not obvious in the sparse area, the interstitium is interspersed with collagen fibers, and the vitreous changes can be seen around the blood vessel wall. **(B)** STAT-6M marker: diffuse strong positive expression of tumor cell nuclei.

Pathological analysis confirmed the presence of a solitary fibrous tumor, characterized by histomorphology and immunophenotype (**Figures 2A, B**), including CD34 (+), SATA6 (+), Vimentin (+), and Ki-67 (+, about 3%). The Patient had no postoperative functional deficits. Post-operative imaging showed no definite tumor tissue, which meant that the tumor was completely removed. According to the 2021 World Health Organization (WHO) classification of central nervous system tumors (Fifth Edition), the patient has a WHO grade 3. The patient was observed for 6 months after treatment, with good healing of the wound, no complaints of special symptoms, and no tumor relapse as suggested by MRI.

## Discussion

ISFT is rare, with only a few reports reported in the literature, and it usually presents as a spindle cell tumor. ISFT is predominantly found near the base of the skull, sagittal sinus (6), pars cerebralis, cerebellar vermis, and venous sinus. Most ISFT are solitary, which may occur as multiple lesions. Generally, ISFT is considered a benign tumor, with only 10–20% presenting malignant or potentially malignant features. The clinical manifestations are mostly dependent on the location of tumor development and may be characterized by tumor-occupying symptoms and intracranial hypertension (7). Stout and Murray proposed the hemangiopericytoma cell tumor (HPC) in 1942. Based on the 2007 World Health Organization (WHO) classification of central nervous system tumors, HPC and SFT were initially separate lesions. Owing to their similarity in genetic characteristics to HPC, until 2016 WHO named them jointly as a disease called SFT/HPC. According to the 2021 World Health Organization (WHO) classification of central nervous system tumors (Fifth Edition), the name HPC has been deleted and is now generally called SFT and graded as grades 1 ~ 3 (8).

The ISFT reported in this case had a predominantly cystic component, and previous case reports of cystic degeneration of the ISFT have been reported in the literature (9), but the low prevalence and the lack of specificity in imaging make it challenging to differentiate from other intracranial tumors. In this paper, we report a case of an intracranial solitary fibroma with a predominantly cystic component in a patient who sustained a left occipital lobe cerebral contusion due to an accidental fall a few years ago, which was similar to the area where the tumor occurred, and which we speculated might be related to the tumor’s development. The patient’s previous cranial CT showed multiple cystic segregation-like hypodensity shadows with well-defined lesions. Generally, on CT, ISFT presents as an irregular, oval, well-defined extracerebral tumor, which is mainly manifested as equidensity to hyperdensity, which may be related to the different tissue compositions; Hemorrhage, necrosis, and cystic degeneration are seen in some of the tumors, and calcification is rare; it may be accompanied by erosive destruction of adjacent cranial bones without osteosclerosis (10). Enhanced scans show significant enhancement of the parenchymal part of the tumor, without significant enhancement of necrosis and cystic degeneration within it. On MRI, T_1_WI showed multiple cystic hypointensity masses with the T_2_WI showing mixed hyperintensity, combined with previous literature of dense collagen fibers and sparse spindle cells showing hypointensity, and areas of spindle cell aggregation showing slightly hypersignal. In this situation, the long T_2_ signal of the mass implies that ISFT is a blood-rich tumor with rapid tumor growth or erosion of peripheral blood vessels and insufficient neovascular supply, leading to cystic degeneration and necrosis of the tumor due to lack of oxygen and hypoxia, with unrestricted diffusion. Enhanced T_1_WI scans show marked enhancement of a rounded hypersignal area, known as the “black-and-white reversal sign”, which is characteristic of this condition (11). DSA showed irregular vascular array of the tumor, most of it received bilateral blood contribution and could receive blood contribution from internal and external carotid arteries and their branches or even vertebral basilar artery, which manifested as moderate to highly neovascularized tumor.

Diagnosis of ISFT is mainly based on pathological or immunohistochemical findings, where ISFT is usually composed of spindle-shaped cells that are tightly arranged and spaced with fibrous tissue proliferation and collagenization. The tumor cells are sparsely packed with no specific histone expression. For immunohistochemistry, Schweizer et al. in 2013 described the genotype of NAB2-STAT6 as the most specific diagnostic marker, with a sensitivity and specificity of 97% (12). Up to now, immunohistochemical staining has usually shown that tumor cells differentially express CD34, BCL-2, wave proteins and CD99, which are almost always positively or strongly positively expressed in SFT (13).

ISFT is easily misdiagnosed due to the non-specific nature of its imaging presentation. It usually needs to be differentiated from the following diseases: ① Cystic meningioma: Cystic meningiomas are common in middle-aged and elderly women. The parenchymal part of cystic meningioma has the same imaging manifestations as typical meningioma, with long T_1_ and long T_2_ signals on MRI, which are connected to the meninges in a broad base, which is the typical “meningocele sign” (14–16), and the adjacent bone changes are mainly hyperplasia and sclerosis, and erosive bone destruction is uncommon. ② Hemangioblastoma (HB), which is a benign tumor of vascular origin, accounts for approximately 2% of intracranial tumors and is more common in adults (17), frequently located in the cerebellum. Typical imaging of hemangioblastoma presenting as a large capsule with small nodules is a T_1_WI enhancement scan suggesting marked enhancement of the wall nodules, with little enhancement of the capsule wall if a cystic component is present; abnormally expanding vascular shadows can be seen in the tumor and around the tumor. ③ Pilocytic astrocytoma, the most common pediatric glioma, in which the cerebellum is the most common area, presents as a cystic solid mass with no or little peritumoural edema, with significant enhancement of the solid component on enhanced scan.

Surgical resection is the first-line treatment for SFT. As the blood supply of the tumor is adequate, resection of ISFT may result in significant intraoperative bleeding, and preoperative embolization (18) may reduce the risk of intraoperative bleeding and improve resectability. As the knowledge of the aggressive biology of ISFT and the parameters of tolerance-assisted stereotactic radiosurgery (SRS) improves (19), SRS should be used as early as possible for postoperative treatment. Nowadays, studies have shown (20) that mutations in IDH1 p.R132S and PD-L1 expression have been identified as potential therapeutic targets for SFT, providing more precise options for clinical treatment.

SFT is classified into three grades in the 2021 WHO classification system, with grade II and III patients being more likely to be malignant because they have higher rates of relapse, extracranial metastases, and mortality. It is reported in that the surgical resection as a first-line treatment option, followed by adjuvant radiotherapy after resection improves survival, and recent studies have shown that the 5-year survival rate of ISFT has improved significantly, although there is no difference in overall survival, the local control rate has increased from 60% to 90%; CS prediction was visualized for dynamic changes in patient survival probability, which greatly alleviated patient anxiety and facilitated doctor-patient communication (21). Postoperatively, patients should be advised to undergo MRI follow-up at six months intervals to pay careful attention to the changes in the patient’s disease course, and promptly diagnose and treat changes in the patient’s condition.

## Conclusion

Cystic intracranial solitary fibroma is a rare intracranial tumor with a lack of specificity in its imaging presentation. This article presents the case of a 42-year-old male patient with a cystic intracranial solitary fibroma of the left occipital region, which on magnetic resonance showed a multifocal atrial cystic mass growing across the cerebellar tegmentum, with the fourth ventricle markedly displaced by compression, and with no enhancement of the cystic component and marked enhancement of the cystic wall on enhancement scans. Currently, pathology and immunohistochemistry are the gold standard for its diagnosis. This case report aims to improve the understanding and knowledge of cystic intracranial solitary fibrous tumor.

## Data availability statement

The raw data supporting the conclusions of this article will be made available by the authors, without undue reservation.

## Ethics statement

Written informed consent was obtained from the individual(s) for the publication of any potentially identifiable images or data included in this article.

## Author contributions

YL: Writing – original draft. LJ: Writing – review & editing. DL: Writing – review & editing. LY: Writing – review & editing. JZ: Writing – review & editing. XG: Writing – review & editing. LS: Writing – review & editing. BT: Writing – review & editing. TL: Writing – review & editing.
